# *LEAFY* and Polar Auxin Transport Coordinately Regulate *Arabidopsis* Flower Development

**DOI:** 10.3390/plants3020251

**Published:** 2014-04-30

**Authors:** Nobutoshi Yamaguchi, Miin-Feng Wu, Cara M. Winter, Doris Wagner

**Affiliations:** 1 Department of Biology, University of Pennsylvania, Philadelphia, PA 19104, USA; E-Mails: nobuy@sas.upenn.edu (N.Y.); miin@sas.upenn.edu (M.-F.W.); 2 Department of Biology, Duke University, Box 90338, Durham, NC 27708, USA; E-Mail: cara.winter@duke.edu

**Keywords:** *Arabidopsis thaliana*, auxin transport, CUP-SHAPED COTYLEDON2, ETTIN, flower development, LEAFY, PIN-FORMED1, PINOID

## Abstract

The plant specific transcription factor LEAFY (LFY) plays a pivotal role in the developmental switch to floral meristem identity in *Arabidopsis*. Our recent study revealed that LFY additionally acts downstream of AUXIN RESPONSE FACTOR5/MONOPTEROS to promote flower primordium initiation. LFY also promotes initiation of the floral organ and floral organ identity. To further investigate the interplay between LFY and auxin during flower development, we examined the phenotypic consequence of disrupting polar auxin transport in *lfy* mutants by genetic means. Plants with compromised LFY activity exhibit increased sensitivity to disruption of polar auxin transport. Compromised polar auxin transport activity in the *lfy* mutant background resulted in formation of fewer floral organs, abnormal gynoecium development, and fused sepals. In agreement with these observations, expression of the auxin response reporter *DR5rev::GFP* as well as of the direct LFY target *CUP-SHAPED COTYLEDON2* were altered in *lfy* mutant flowers. We also uncovered reduced expression of *ETTIN*, a regulator of gynoecium development and a direct LFY target. Our results suggest that *LFY* and polar auxin transport coordinately modulate flower development by regulating genes required for elaboration of the floral organs.

## 1. Introduction

The phytohormone auxin is a central regulator of lateral organ initiation [1,2,3,4]. Auxin accumulates in a graded and dynamic manner with the sites of auxin maxima correlating with the sites of primordium initiation [3,5]. The formation of auxin gradients is established by local auxin biosynthesis and polar auxin transport [1,2,3,4,5,6,7,8]. Direct transport is controlled by the PINFORMED (PIN) proteins, which encode auxin efflux carriers and exhibit polarized plasma membrane localization [1,2,3,4,5]. The Ser/Thr protein kinase PINOID (PID) catalyzes PIN phosphorylation and contributes to the regulation of apical-basal PIN polarity [9,10]. *NAKED PINS IN YUC MUTANT* (*NPY*) family genes, which encode NONPHOTOTROPIC HYPOCOTYL 3-like proteins, regulate PIN endocytosis and control auxin accumulation in incipient organ primordia [11,12,13]. On the other hand, YUCCA (YUC) flavin monooxygenase catalyzes a rate-limiting step in a tryptophan-dependent auxin biosynthesis pathway [14,15,16]. Mutations in *PIN1*, *PINOID*, *NPY*, and *YUC* result in inflorescences that form only a few flowers and grow as a pin-like structure [1,12,16,17].

Subsequent to determination of the primordium initiation site by an auxin maximum, the incipient flower primordium undergoes extensive growth. The AUXIN RESPONSE FACTOR5/MONOPTEROS (ARF5/MP) has a central role translating local auxin concentration into specific gene expression outputs and flower initiation [18,19]. In the absence of auxin, MP activity is inhibited by the physical interaction between MP and Aux/IAA proteins such as BODENLOS/IAA12 [20,21], this represses transcription of downstream target genes involved in flower formation. Auxin sensing promotes the degradation of BDL/IAA12, resulting in MP-dependent transcriptional activation of target genes [20,21,22]. MP directly binds to the regulatory region of *LEAFY* (*LFY*), which specifies floral fate [23,24], and to two transcription factors, *AINTEGUMENTA* and *AINTEGUMENTA-LIKE6/PLETHORA3* (*AIL6/PLT3*), key regulators of floral meristem outgrowth [25,26,27,28]. The *lfy ant ail6* triple mutant is defective in flower primordium initiation, while reintroduction of LFY and ANT activity into *mp* mutants partially rescues the organogenesis defect [22]. These results suggest that upregulation of *LFY*, *ANT*, and *AIL6/PLT3* by MP contributes to flower primordium initiation. 

In addition to regulating floral primordium initiation, auxin also regulates floral organ development [29]. Plant harboring mutations in *PIN1*, *PID*, *YUC*, and *NPY* produce a few abnormal flowers [1,12,16,17]. These flowers typically have fewer sepals and stamens, more petals, fused floral organs, and valveless gynoecia. The observed alterations in gynoecium patterning are similar to those resulting from mutations in *ETTIN* (*ETT*), which encodes AUXIN RESPONSE FACTOR3, or from treatment with an auxin transport inhibitor [30]. Floral organ fusion phenotypes are caused by loss-of-function mutations in the *CUP-SHAPED COTYLEDON* (*CUC*) genes [31,32]. *lfy* single mutants do not display any defects in floral primordium initiation or in floral organ development, similarly to *pin* or *pid* mutants [1,17,23]. However, LFY interacts genetically with PID in lateral organ primordium initiation [33]. 

Here, we further probe the interactions between LFY and polar auxin transport. We have examined the consequences of loss-of-LFY function in plants compromised in polar auxin transport by genetic means. These experiments demonstrate that *LFY* promotes not only flower primordium initiation, but also subsequent floral organ initiation and development, in concert with polar auxin transport. LFY executes this role likely via regulating downstream direct targets, such as *CUC2* and *ETT*, whose activity is also controlled by polar auxin transport. Our study uncovers a complex set of interactions between LFY and the auxin pathway in flower development.

## 2. Results and Discussion

### 2.1. Loss-of-LFY Function Enhances Floral Primordium Initiation Defects of pin1 and pid Mutants

**Figure 1:**
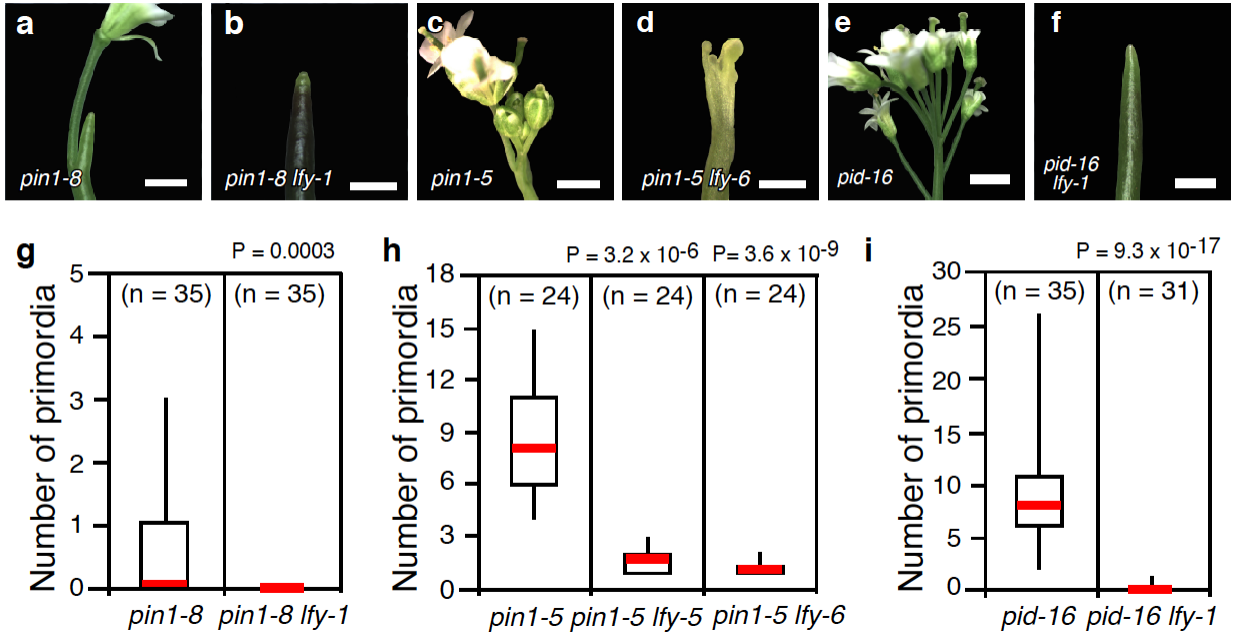
*lfy* enhances the floral primordium initiation defects of auxin transport mutants. (**a**–**f**) Close-up view of the inflorescences formed in a strong *pin1* mutant allele, *pin1-8* (**a**); and *pin1-8* *lfy-1* (**b**); a weak *pin1* mutant allele, *pin1-5* (**c**); and *pin1-5 lfy-6* (**d**); *pid-16* (**e**) and *pid-16 lfy-6* (**f**); (**g**–**i**) Quantification of the flower initiation defects in *pin1-8* and *pin1-8* *lfy-1* (**g**); *pin1-5*, *pin1-5 lfy-5*, and *pin1-5* *lfy-6* (**h**); and *pid-16* and *pid-16 lfy-6* (**i**)*.* Note that *lfy-1* and *lfy-6* carry the exact same mutation (Q32stop) but are the Columbia (Col) and Landsberg *erecta* (L*er*) cultivar, respectively. We employ both alleles as to eliminate mixed genetic backgrounds when generating double mutants. Scale bar, 200 µm (**a**,**b**,**d**,**f**), 5 mm (**c**,**e**).

Because *pin1* and *pid* single mutants make several flowers, they provide a sensitized background in which to study the interplay between *LFY* and auxin transport during flower primordium initiation. We first introduced the *lfy-1* (Col) null mutant into *pin1* mutants. The strong *pin1-8* allele formed naked inflorescences with a few flowers, as reported previously (Figure 1a,g) [1,34]. In *pin1-8 lfy-1* double mutants, no flowers were produced (Figure 1b,g; *p* < 10^−3^). By contrast, the weak *pin1-5* allele formed approximately 8 flowers per inflorescence, as described previously (Figure 1c,h) [17]. While flowers were produced in double mutants between *pin1-5* and the weak *lfy-5* or *lfy-6* (L*er*) null mutants, they were dramatically reduced in number compared to the *pin1-5* single mutant (Figure 1d,h). These genetic interactions suggest that *LFY* acts synergistically with polar auxin transport to promote flower primordium initiation. 

We next investigated the effect of introducing *lfy-1* into *pid* mutant background using the strong *pid-16* allele (Figure 1e,i). *pid-16 lfy-1* produced only a few flowers compared to the *pid-16* single mutant (Figure 1i; (*p* < 10^−16^)). Taken together, the inability of *lfy* mutants to initiate flower primordia in genetic backgrounds defective in polar auxin transport confirms the proposed role for LFY in auxin-mediated flower primordium initiation [22]. Consistent with this finding, a recent genetic screen identified a *pid* mutation as a second site enhancer of weak *lfy-5* mutants [33]. 

Previously, it has been reported that pin-like apices of *pin1* single and *pin1* *lfy* double mutants retain auxin responsiveness with respect to formation of lateral organs such as flowers and cauline leaves in the inflorescence [2,4]. By contrast, strong *mp* mutants are no longer responsive to auxin [2]. These findings are consistent with the observation that additional direct MP targets act in parallel with LFY in flower primordium initiation [22]. Our data are consistent with the strikingly enhanced defects in lateral organ initiation observed in *mp pid* or *mp pin* double mutants [35], which suggest that MP and MP targets like LFY act, in part, in parallel with these regulators of polar auxin transport. 

### 2.2. Mutations in LFY Enhance Floral Organ Initiation and Growth Defects of pin1 Mutants

**Figure 2:**
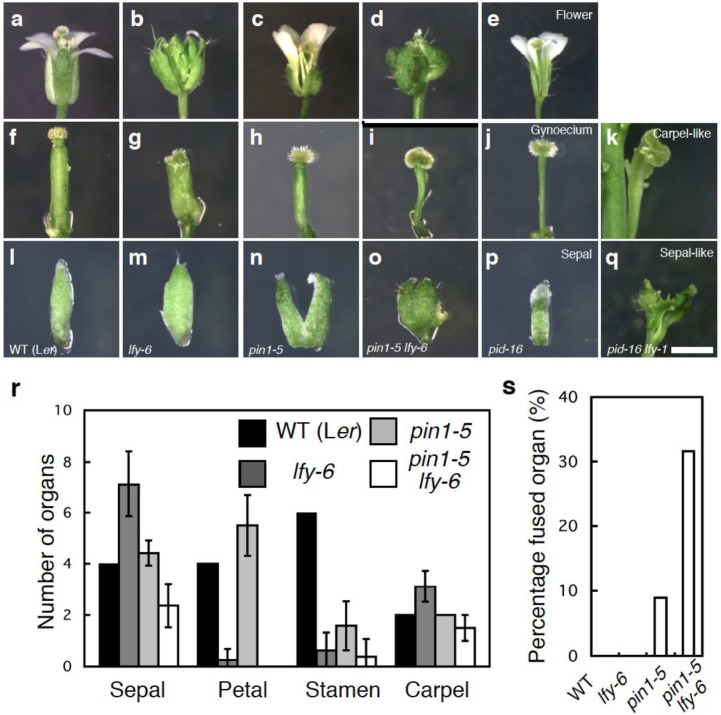
*lfy* enhances the flower developmental defects of auxin transport mutants. (**a**–**e**) Side view of the flowers formed in wild type (**a**), *lfy-6* (**b**), *pin1-5* (**c**), *pin1-5 lfy-6* (**d**), *pid-16* (**e**); (**f**–**k**) Side view of the gynoecium formed in wild type (**f**), *lfy-6* (**g**), *pin1-5* (**h**), *pin1-5 lfy-6* (**i**), *pid-16* (**j**), *pid-16 lfy-1* (**k**); (**l**–**q**) Side view of the sepal formed in wild type (**l**), *lfy-6* (**m**), *pin1-5* (**n**), *pin1-5 lfy-6* (**o**), *pid-16* (**p**), *pid-16 lfy-1* (**q**); (**r**) Floral organ number in wild-type, *lfy-6*, *pin1-5*, and *lfy-6 pin1-5* flowers; (**s**) Incidence of sepal fusion in wild-type, *lfy-6*, *pin1-5*, and *lfy-6 pin1-5* flowers. Scale bar, 2 mm.

Because only *pin1-5 lfy-6* inflorescences make a reasonable number of flowers prior to a naked pin, we first examined the effect of loss of *LFY* function on floral organ development in the *pin1-5* mutant. Floral organs in wild-type plants are arranged in a series of whorls: four sepals in the outer whorl, followed by four petals, six stamens, and two carpels (Figure 2a,r). As reported previously, *lfy* mutant flowers display floral homeotic defects. *lfy* null mutants rarely form petals and stamens and largely consist of sepal- and carpel-like organs [23] (Figure 2b,r). This is consistent with molecular and genetic data, which show that LFY plays a major role in the up-regulation of the class B and class C floral homeotic genes [36,37,38]. On the other hand, the weak *pin1-5* flowers formed all four types of floral organs, like wild-type flowers (Figure 2c,r). However, *pin1-5* flowers showed an increase in the number of petals, and a decrease in the number of stamens formed (Figure 2r) [17]. In addition, *pin1-5* resulted in a reduction of the valve region and expansion of the stylar and stigmatic regions of the gynoecium (Figure 2f–h). Finally, *pin1-5* sepals are often fused (Figure 2l–n). These phenotypes are common in flowers of auxin defective mutants [15,16,30]. 

*pin1-5 lfy-6* exhibited a reduction in the number of all four floral organs formed, compared with the wild-type or parental lines and generally lacked petals and stamens (Figure 2d,r). The sepal fusion and abnormal gynoecium phenotype of *pin1-5* was enhanced in the double *pin1-5 lfy-6* mutants (Figure 2h,i,n,o). The length of the fused sepal margin increased when compared with *pin1-5* single mutant flowers. The incidence of sepal fusion also increased in *pin1-5 lfy-6* flowers (32%) compared to *pin1-5* flowers (8.9%) (Figure 2s). In individual plants, sepal fusions became more severe and frequent acropetally (Figure 2n,o). Additionally, the size of the stigmatic and ovary regions in the gynoecium increased and decreased, respectively when compared with *pin1-5* single mutant flowers (Figure 2h,i). Although *pin1-5* formed a few seeds, *pin1-5 lfy-6* flowers were infertile, like *lfy-6* mutants (data not shown). 

To further confirm the role of *LFY* in auxin transport-mediated flower development, we examined genetic interaction between *LFY* and *PID*. *pid-16* single mutants produced several flowers with fewer floral organs (Figure 2e). The flowers produced by *pid-16 lfy-1* plants exhibited more severe phenotypic defects than those observed in *pid-16* alone (Figure 2e,j,k,p,q). *pid-16 lfy-1* flowers never formed petals and stamens (Figure 2k). Flowers of *pid-16 lfy-1* double mutants were comprised of either several fused carpel-like or sepal-like organs (Figure 2k,q). In addition these abnormal flowers often displayed fusion with the pedicel (Figure 2k,q). Thus, *LFY* may act together with polar auxin transport in sepal and gynoecium development.

### 2.3. The Relationship between LFY and Auxin-Mediated Sepal Initiation

**Figure 3:**
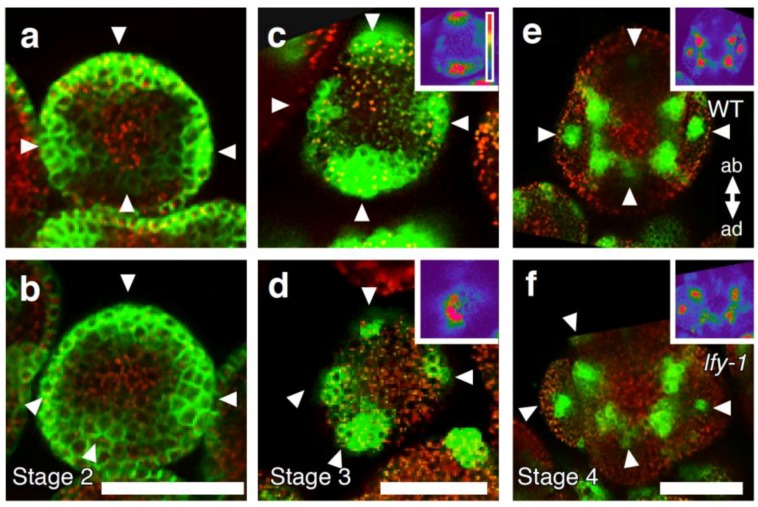
Floral organ primordium initiation in *lfy* loss-of-function mutant flowers. (**a**,**b**) pPIN1::PIN1-GFP expression in wild-type (**a**) and *lfy* (**b**) flowers at stage 2. (**c**–**f**) *DR5rev::GFP* expression in wild-type (**c**,**e**) and *lfy* (**d**,**f**) flowers at stage 3 (**c**,**d**) and stage 4 (**e**,**f**). Strongly increased GFP signal was observed in sepal primordia (arrowheads), as reported previously [3,39]. Insets show color-coded GFP intensity, with background levels being purple and blue, green, yellow, and red to signifying increasing *DR5rev::GFP* signal. ab, abaxial; ad, adaxial. Scale bar, 25 µm.

The sepal phenotype observed in *pin1 lfy* flowers may be due to an alteration in the positioning of sepal primordia in the floral meristem and/or the inability to establish boundaries between adjacent organs. To better understand how loss of *LFY* function affects sepal formation, we examined sepal primordium positioning by monitoring expression of the auxin efflux carrier protein, PIN1, and the auxin response promoter *DR5*. In stage 2 wild-type flowers, PIN1-GFP was expressed in the incipient sepal primordia at symmetrical positions within the flower meristem (Figure 3a). The PIN1-GFP positive sites of sepal primordium initiation in stage 2 flowers of *lfy* mutants were not properly spaced, as in the wild type. Frequently two incipient sepal primordia formed in close proximity (Figure 3a,b). In wild-type plants, *DR5rev::GFP* was expressed in the sepal primordia, as reported previously [3,39]. On the basis of *DR5rev::GFP*, wild-type sepals developed four discrete sepal primordia at symmetrical positions (Figure 3c). Like *lfy* floral primordia [22,33], *lfy* sepal primordia showed slightly reduced *DR5* expression, especially at the abaxial and the lateral side of the sepals (Figure 3c,d insets). Although we saw an increase in the number of sepals in *lfy* mutants at later stages, stage 3 *lfy-1* mutant flowers only had four sepal primordia based on *DR5* expression (Figure 3c,d). These data suggest that additional sepal primordia form after stage 3 in *lfy* flowers. As seen for the PIN1-GFP expression, the sites of sepal initiation marked by *DR5* in stage 3 flowers of *lfy* mutants were not properly spaced in the floral primordium; frequently two sepal primordia formed in close proximity (Figure 3b,d). At stage 4 of flower development, *DR5* expression in *lfy* sepals was often not perfectly cruciform, but somewhat twisted (Figure 3e,f). Hence, LFY is required for proper positioning of sepal primordia.

### 2.4. A Link between LFY and Polar Auxin Transport in Sepal Boundary Formation

**Figure 4:**
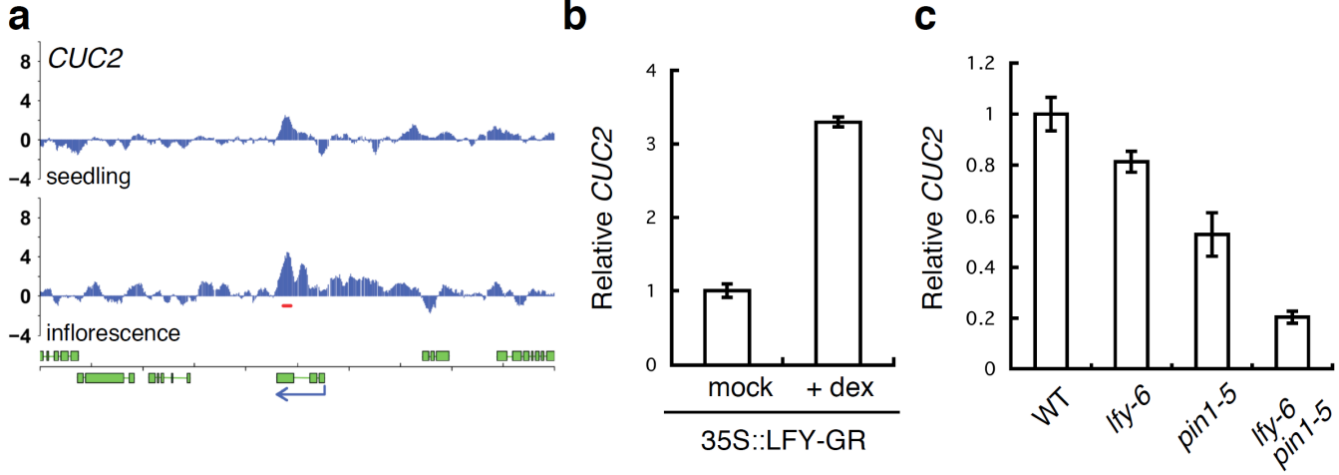
LFY regulates *CUC2* gene expression. (**a**) LFY binds to the regulatory regions of *CUC2* gene based on a published ChIP-chip data set [37]. Screenshots of the binding peaks (blue vertical lines). Significant LFY-binding peaks are indicated by a red horizontal line below the peaks. Green boxes below the graphs denote exons, blue arrows indicate the direction of transcription; (**b**) Expression of the *CUC2* gene based on qRT-PCR three hours after mock or dexamethasone (dex) treatment of *lfy-6* 35S::LFY-GR inflorescences; (**c**) Expression of the *CUC2* gene based on qRT-PCR in *lfy-6* null mutant, the weak *pin1-5* mutant, and *pin1-5 lfy-6* double mutant inflorescence apices compared to wild-type inflorescence apices. RNA from floral bud clusters (containing stages 1–6 flowers) was used in this study.

A further connection between LFY and sepal development was suggested by genome-wide identification of LFY binding sites [37,40]. The most highly enriched Gene Ontology terms among direct LFY targets in the inflorescence were “organ development” and “flower development” [37,40]. Amongst the candidate direct LFY targets identified by these studies, one gene with a known role in organ boundary formation stood out, *CUC2*. Although LFY is already expressed prior to flower formation [41], LFY only bound to the 5'-regulatory region of the *CUC2* gene in inflorescences (Figure 4a), suggesting that LFY regulates *CUC2* expression specifically at this stage. We further found that *CUC2* mRNA levels rapidly increased upon 35S::LFY-GR activation in inflorescences (Figure 4b). In addition, *CUC2* showed a subtle, but reproducible reduction of expression in *lfy-6* null mutants relative to the wild type (Figure 4c). *CUC2* mRNA levels were also reduced in *pin1-5* mutants. Furthermore, a more dramatic reduction in *CUC2* expression was observed in *pin1-5 lfy-6* compared to the wild type or the parental lines, suggesting that both *LFY* and auxin transport are important for proper *CUC2* induction in the flower. 

**Figure 5:**
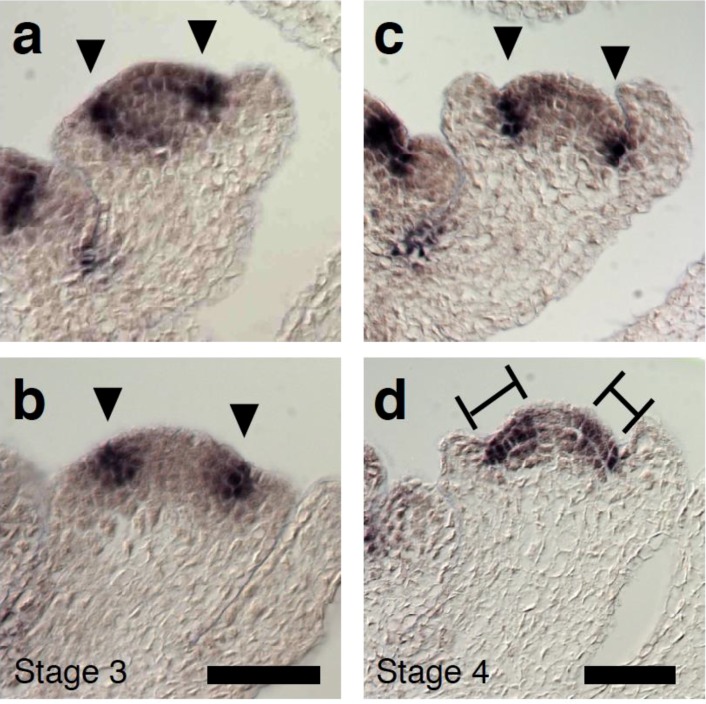
Altered *CUC2* gene expression in *LFY* loss-of-function flowers. (**a**–**d**) *CUC2* expression in wild-type (**a**,**c**) and *lfy* (**b**,**d**) flowers at stage 3 (**a**,**b**) and stage 4 (**c**,**d**). Arrowheads denote the boundary between the floral meristem and the sepals. Bars indicate diffused *CUC2* expression. Scale bar, 25 µm.

To further test the relationship between LFY and *CUC2*, we performed *in situ* hybridization. In wild-type plants, *CUC2* was expressed in the sepal boundary of stage 3 flowers, as reported for *CUC1* or *CUC3* (Figure 5a) [42,43]. *CUC2* expression pattern and levels were essentially the same in stage 3 wild type and *lfy-1* flowers (Figure 5b). In stage 4 wild-type flowers, strong *CUC2* expression was observed in the boundary region between the sepal primordia and the floral meristem dome, where growth is retarded (Figure 5c,e). However, *CUC2* expression in the boundary between the sepal primordia and the floral meristem dome was frequently weaker in stage 4 *lfy-1* flowers and *CUC2* expression was overall more diffuse (Figure 5d). Occasionally, *CUC2* expression was entirely missing from a boundary region (Appendix Figure A1). 

It has been reported that *CUC1* and *CUC2* genes act redundantly in regulating formation of the sepal boundary [31], and that *CUC2* expression is affected by auxin transport [44]. Since we could observe both a sepal fusion phenotype and a dramatic reduction of *CUC2* mRNA levels in *pin1 lfy* double mutants, the combined data are consistent with the idea that LFY and polar auxin transport may modulate *CUC2* expression in the sepal boundary.

### 2.5. A Link between LFY and Auxin Transport in Gynoecium Development

**Figure 6:**
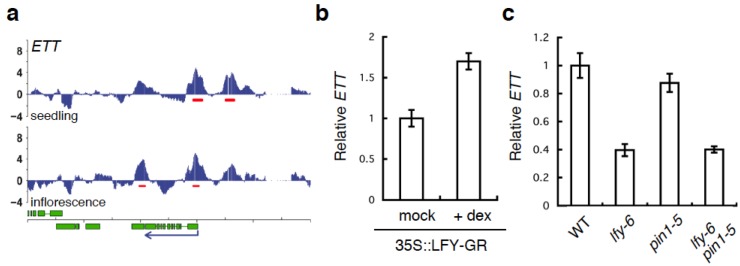
LFY regulates the *ETT* gene. (**a**) LFY binds to the regulatory regions of the *ETT* gene based on a published ChIP-chip data set [37]. Screenshots of the LFY binding peaks (blue vertical lines). Significant LFY-binding peaks are indicated by a red horizontal line below the peaks. Green boxes below the graphs denote exons, blue arrows indicate the direction of transcription; (**b**) Expression of the *ETT* gene based on qRT-PCR three hours after mock or dexamethasone (dex) treatment of *lfy-6* 35S::LFY-GR inflorescences (**c**) Expression of the *ETT* gene based on qRT-PCR in *lfy-6* null mutant, weak *pin1-5* mutant, and *pin1-5 lfy-6* double mutant inflorescence apices compared to wild-type inflorescence apices. RNA from floral bud clusters (containing stages 1-6 flowers) was used in this study.

Among the LFY-bound genes identified by ChIP-Seq or ChIP-chip are many genes in the auxin pathway [22,37,40], several of which have been implicated in gynoecium development [12,15,30]. Thus, several potential direct LFY target genes may underlie the gynoecium formation defects observed in *pin lfy* mutants. Because the single *ett* null mutant displays gynoecium defects very similar to those we observed here [30], we focused on the relationship between LFY and *ETT*. Two strong LFY binding peaks were present at the *ETT* locus in inflorescences, one in the promoter and one in the ninth intron (Figure 6a). When we tested *ETT* expression by qRT-PCR, we found that *ETT* mRNA was reduced in *lfy* mutants and rapidly increased upon 35S::LFY-GR activation in inflorescences (Figure 6b,c). These results suggest that LFY may activate *ETT* to regulate gynoecium development. However, we did not see a strong reduction of *ETT* expression in *pin1-5* mutants compared to wild type (Figure 6c). Moreover, no further change in *ETT* expression was observed in *pin1-5 lfy-6* mutant relative to *lfy-6* mutant inflorescences (Figure 6c). Thus, additional regulators of gynoecium development besides *ETT* may contribute to the developmental defects observed in *pin1 lfy* double mutants. 

Alternatively, reduced *ETT* accumulation in *lfy* mutants combined with reduced ETT activity in *pin1-5* may cause the observed gynoecium defect. Although the ETT protein lacks the conserved domains III and IV for interaction with AUX/IAA proteins [45], it has been reported that the transcriptional repressor ETT suppresses a synthetic AuxRE-based reporter in protoplasts in an auxin-dependent manner [46], and *pin1* mutants have reduced auxin accumulation [1]. In agreement with this study, treatment with the auxin transport inhibitor NPA enhanced the gynoecium defects of weak *ett* mutants to phenocopy *ett* null mutants, while *ett* null mutant gynoecium defects were not enhanced by this treatment [30]. Thus, we favor a model in which the reduction of *ETT* mRNA level (due to the *lfy* mutation) and attenuated ETT protein activity (due to the *pin1* mutation/low auxin level) cause gynoecium developmental defects in *pin1 lfy* double mutants. Further studies are required to resolve how auxin modulates ETT activity in the gynoecium.

## 3. Experimental Section

### 3.1. Plant Materials and Growth Conditions

Plants were grown at 23 °C in a 16 h light/8 h dark cycle. *Arabidopsis thaliana* accessions Columbia (Col) or Landsberg *erecta* (L*er*) were used in this study. Mutants employed were: *lfy-1* [23], *lfy-6* [23], *pin1-5* [17], *pin1-8* (SALK_97114) [34], *pid-16* (SALK_082564), 35S::LFY-GR [47], and *DR5rev::GFP* [3]. For dexamethasone treatments, plants were grown in soil. Dexamethasone treatments were performed by spraying 30-day-old plants once with 5 µM dexamethasone.

### 3.2. Statistical Tests

The Student *t*-test (two-tailed) was used for all experiments that displayed a normal distribution based on the Kolmogorov–Smirnov test [48].

### 3.3. Genetics and PCR Genotyping

*lfy/+* plants were crossed to *pin1-5*, *pin1-8/+*, or *pid-16/+* plants. Double mutants were identified in the F2 or later generations as plants with new phenotypes and confirmed by PCR genotyping. *lfy* mutations were PCR genotyped as described previously. Genotyping primers are shown in Table 1.

**Table 1:** Primer sets used in this study.

Primer name	Sequence
**Genotyping**	
*lfy-1/-6-*FW	AAGCAGCCGTCTGCGGTGTCAGCAGCTGTT
*lfy-1/-6*-RV	CTGTCAATTTCCCAGCAAGACAC
*pid-16*-LP	TCCGTCATAGACAACCTCACC
*pid-16*-RP	GAGTAAGCGTACGAATGAGCG
*pin1-8*-LP	AACTGGCTTCACAGCAGAAAG
*pin1-8*-RP	TCAACAAAAAGGGCATTGTTC
LBb1.3	ATTTTGCCGATTTCGGAAC
**Primer name**	**Sequence**
**qRT-PCR**	
CUC2-FW	GGAAGAGCTCCGAAAGGAGA
CUC2-RV	TCCGGTGCTAGCTAAAGTGG
ETT-FW	CAGGGACATTTGGAACAAGC
ETT-RV	CAGGAAGAAGAGAGACTTGAGCA
EIF4-FW	AAACTCAATGAAGTACTTGAGGGACA
EIF4-RV	TCTCAAAACCATAAGCATAAATACCC
***In situ* hybridization**	
CUC2-FW	CGGAATTCATGGACATTCCGTATTACCA
CUC2-RV	CCACTAGTTCAGTAGTTCCAAATACAGT

### 3.4. Confocal Microscopy

Imaging of GFP signal was performed as described by [22]. For imaging of GFP signal, inflorescence apices were dissected to remove older flowers and imaged for green and red fluorescence using a Leica confocal microscope (Leica, LCS SL) equipped with an argon-krypton ion laser with the appropriate filter sets for visualizing GFP and propidium iodide. For comparisons of GFP fluorescence, the same offset and gain settings were used in the analysis of plants that were transformed with the same transgene. At least ten inflorescences were prepared from different plants, and representative images are shown.

### 3.5. Quantitative RT-PCR

RNA from floral bud clusters (containing stages 1–6 flowers) was used in this study. Total RNA was extracted from inflorescences using the RNeasy mini kit (Qiagen) and was treated with DNase (Qiagen) prior to RT. cDNA was synthesized from 0.5 ng of total RNA with the Super Script III (Invitrogen). qRT-PCR was performed as previously described by Yamaguchi *et al.* [22]. Real-time PCR was performed with the 7100 Real-Time PCR system and Power SYBR Green PCR Master Mix (Applied Biosystems), as described by the manufacturer. The means and standard errors were determined using two to three biological replicates with three technical replicates each. Plant materials were grown and harvested at different times. One representative experiment is shown. Gene-specific signals were normalized over that of the *EUKARYOTIC TRANSLATION INITIATION FACTOR 4A-1* (*EIF4; At1g54270*). Primers are shown in Table 1.

### 3.6. Screenshot

Significant LFY binding to regulatory regions of *CUC2* and *ETT* in seedlings and inflorescences were generated by Winter *et al.* [37].

### 3.7. In situ Hybridization

*In situ* hybridization was performed as described previously [49,50]. The *CUC2* probe consisted of base pairs 29 to 1137 (TSS = 1). Cloning primers are shown in Table 1. Probes were cloned into pGEM-T Easy (Promega). Antisense *CUC2* probe was digested with *Eco*RI and transcribed with the T7 polymerase. The Riboprobe Combination System (Promega) and DIG RNA labeling mix (Roche) were used for probe synthesis. 

## 4. Conclusions

The plant specific transcription factor LFY is necessary and sufficient for the developmental transition to flower formation [23,24] Previous genomic, genetic, and molecular analyses have suggested a role for LFY downstream of AUXIN RESPONSE FACTOR5/MONOPTEROS in flower primordium initiation [22,37,40]. In addition, LFY feedback regulates the auxin pathway [22,33]. Finally, like MP, LFY also acts in a pathway parallel to the polar auxin transport regulators PID and PIN1 in lateral organ initiation (this study) [33,35]. We have uncovered enhanced sensitivity of *lfy* mutants to alterations in polar auxin transport in other aspects of flower morphogenesis such as floral organ initiation and sepal and gynoecium development. It is difficult to know whether the interactions between auxin transport and LFY are direct, via changes in auxin concentrations, or indirect via combinatorial effects on gene expression. Disruption of polar auxin transport has a dramatic effect on expression of a spectrum of genes [51]. We provide evidence that transcriptional regulation of the organ boundary regulator *CUC2* and the gynoecium development regulator *ETT* by LFY (this study) and auxin [1,2,3,4,5,6,7,8,9,10,11,12,13,14,15,16,17] may contribute to defects in sepal and gynoecium development observed in double mutants between polar auxin transport regulators and *lfy*. Our study combined with prior investigations reveals a complex set of interactions between LFY and polar auxin transport in developing flowers. 

## Figures and Tables

**Figure A1:**
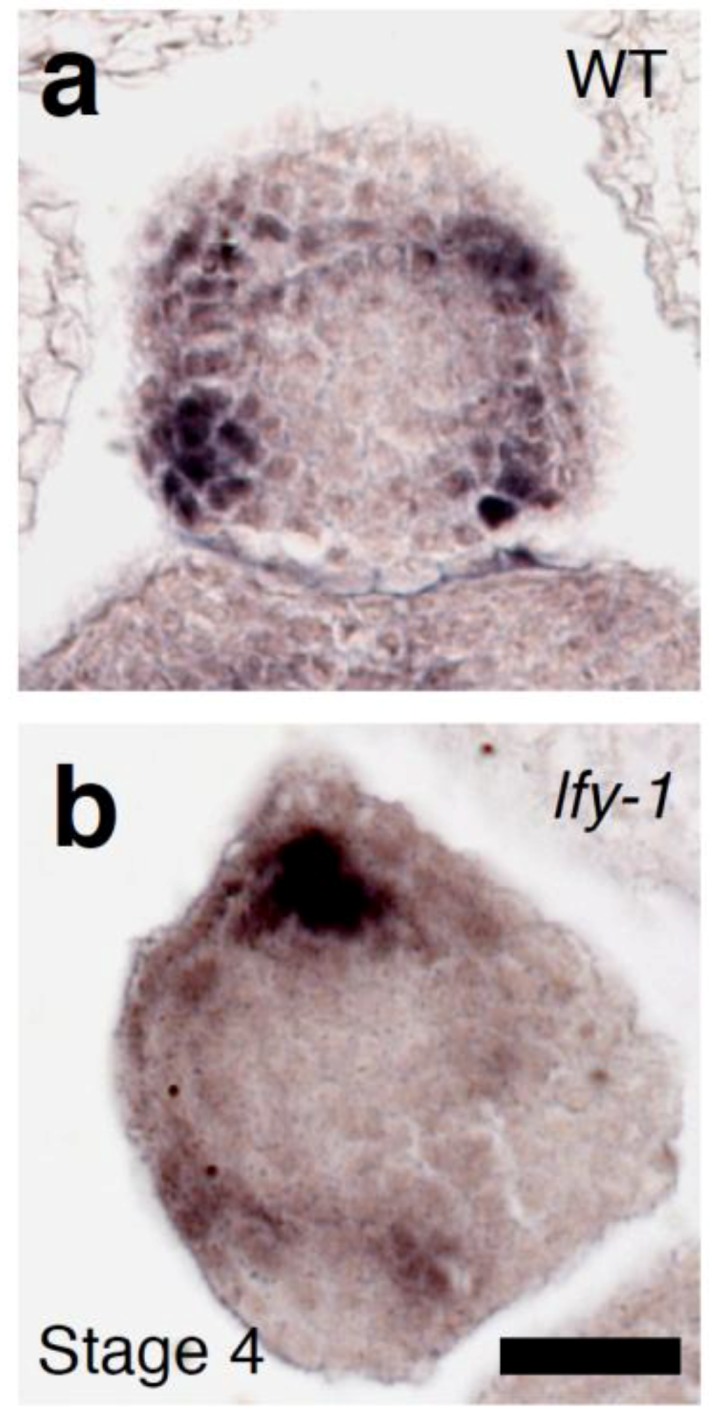
Altered *CUC2* gene expression in *LFY* loss-of-function flowers. (**a**,**b**) *CUC2* expression in wild-type (**a**) and *lfy* (**b**) flowers at stage 4. Arrowheads denote the boundary between the floral meristem and the sepals. Scale bar, 25 µm.
